# Anatomical variants complicating the posterior approaches towards the elbow joint

**DOI:** 10.1007/s00276-023-03124-9

**Published:** 2023-03-15

**Authors:** Vojtech Kunc, Michal Benes, David Veigl, David Kachlik

**Affiliations:** 1 Department of Anatomy, Second Faculty of Medicine, Charles University, V Uvalu 84, 150 06 Prague 5, Czech Republic; 2 Clinic of Trauma Surgery, Masaryk Hospital, Usti nad Labem, Czech Republic; 3 1st Department of Orthopaedics, First Faculty of Medicine, Charles University and University Hospital Motol, Prague, Czech Republic; 4 Department of Health Care Studies, College of Polytechnics, Jihlava, Czech Republic

**Keywords:** Variability around elbow joint, Posterior approach, Dorsoepitrochlearis, Chondroepitrochlearis, Epitrochleoanconeus, Subanconeus, Accessory bones, Ulnar nerve

## Abstract

**Introduction:**

Anatomical variants observed during the posterior approach to the elbow joint require special attention due to their clinical relevance. We aim to present a compendious review of described variants potentially encountered during the posterior approach towards the elbow joint to the experts in the elbow surgery.

**Methods:**

A narrative review of surgical and anatomical textbooks, as well as search of scientific databases was carried out.

**Results:**

Variability of the subcutaneous nerves is important during incision planning. Accessory muscles such as dorsoepitrochlearis, chondroepitrochlearis, epitrochleoanconeus, subanconeus or supernumerary flexor carpi ulnaris may confuse even the senior surgeon during the dissection and possibly complicate the fracture reduction. Some bony variants such as supratrochlear foramen may lead to fracture or possibly interfere with the osteosynthesis placement. Accessory bones are also present in the region of the elbow joint. Those situated intra-articular may present with symptoms.

**Conclusion:**

Many variants can be encountered in the area of the elbow joint and their knowledge is essential to truly understand its anatomy. The presented review enables easier orientation in the current literature with the aim on the posterior approach towards the elbow joint.

## Introduction

Unexpected anatomical variants can make the surgical approach difficult even for experienced surgeons and their detailed knowledge can prevent potential complications. This becomes even more true for junior surgeons who use landmarks for basic orientation that may vary. Many anatomical variants of the cubital region have been published and discussed, but a comprehensive review of their implication and the clinical relevance is still missing.

The posterior approach to the elbow joint (PAEJ) is commonly used for open reduction and internal fixation of the distal part of the humerus, total elbow arthroplasty, removal of loose bodies and treatment of fractures [5, 7]. Thus, profound anatomical knowledge is essential to assure proper and safe treatment of these conditions. Although the unusual morphological appearance of the structures around the elbow may also collide with placement of portals in elbow arthroscopy. Given the clinical importance, the aim of the following text is to review the possible anatomical variants that can be encountered during the PAEJ and other surgical interventions in the area such as the cubital tunnel release.

## Materials and methods

A narrative review of the available literature was performed. Surgical and anatomical textbooks from the authors’ personal collections and academic libraries were initially screened. Consequently, scientific databases including PubMed, Web of Science, and Google Scholar were used to compile additional information on the structures of our interest. No limitations on the literature sources were applied in this study.

## Results

No true internervous plane exists during the PAEJ as the radial nerve enters the triceps muscle proximal to the incision. The standard incision is made 10–15 cm above the olecranon and is slightly curved laterally around the lateral side of the olecranon, which may also allow for access to the proximal radio-ulnar joint. Superficial dissection consists of the palpation, exposition, and subsequent protection of the ulnar nerve in the ulnar nerve groove. Several different techniques for deep dissection are described in the literature and they can be divided into three main categories based on the method of visualization of the articular surface: (1) olecranon osteotomy, (2) triceps reflection, (3) triceps splitting [44]. Nevertheless, most of the described anatomical variants appear during the superficial dissection.

### Variants in superficial dissection

Superficial somatosensory nerves are highly variable at the posterior cubital region and the thoughtless dissection may lead to their damage and subsequent paresthesia or formation of a painful neuroma [38]. Branches of the posterior brachial cutaneous nerve, medial brachial cutaneous nerve, medial antebrachial cutaneous nerve, and the posterior antebrachial cutaneous nerve are in the potential path of the incision. Even though these nerves are significantly variable, a general rule is that four times fewer branches are encountered in the posterior midline compared to the lateral and medial planes [11]. Nevertheless, placement of the incision varies significantly between surgeons. Patients often prefer the scar to be located medially as it is more hidden even though there is a risk of the ulnar nerve irritation. Making the incision more laterally prevents the scar irritation during typing, writing, and drawing. In avoidance of such complication, some surgeons curve the incision laterally around the olecranon. All this should be discussed with the patient to achieve maximal satisfaction.

Immediately after the incision, the ulnar nerve should be identified between the medial intermuscular septum of the arm and the medial head of the triceps brachii muscle. The fourth head of the triceps brachii muscle situated superficial to the medial head could theoretically lead to confusion but to our knowledge only five cases have been described in the literature [6, 14, 18, 42], one of which occurred with several other concomitant variants [35]. All cases reported findings on cadavers and no clinical case has been found in the literature.

**Fig. 1:**
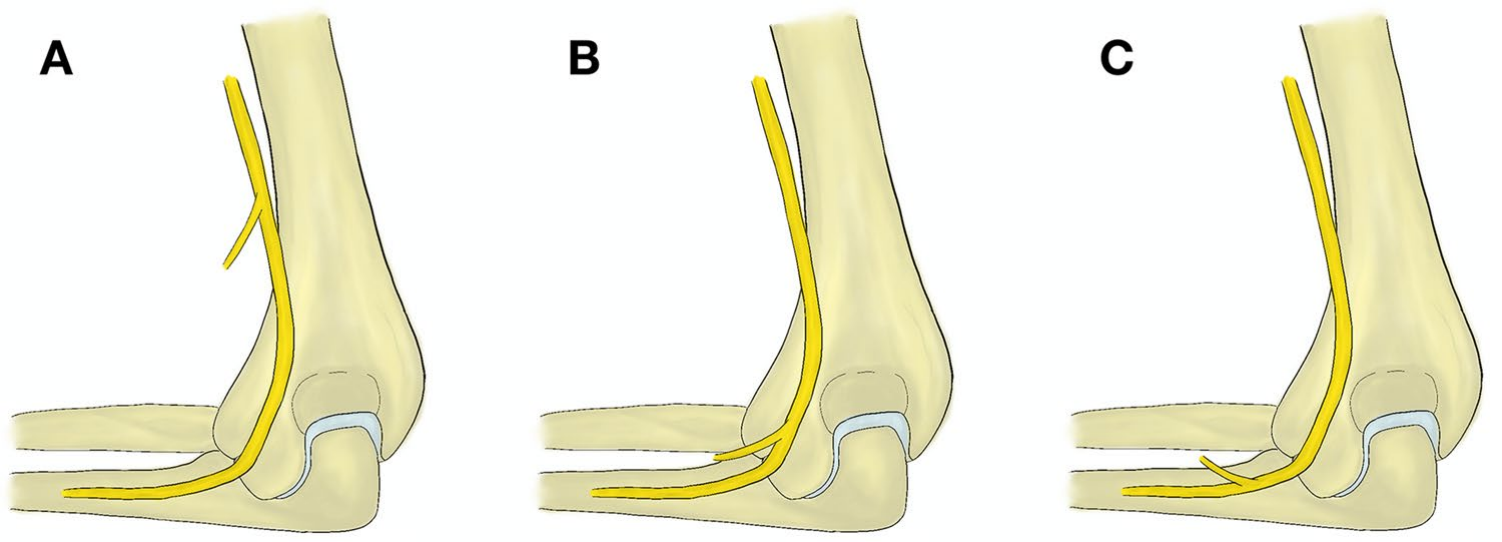
Variable branching of the first branch from the ulnar nerve near the medial epicondyle—**a** proximal to the medial epicondyle, **b** at the condylar level, and **c** below the medial epicondyle

It is necessary to protect the nerve and limit its devascularization. Motor branches usually leave the ulnar nerve below the tip of the medial epicondyle of the humerus. Rarely, the branches for the flexor carpi ulnaris or flexor digitorum profundus muscles arise above the elbow joint and can be therefore injured by the approach (Fig. 1) [22]. A branch to the flexor carpi ulnaris muscle (FCU) was described to usually arise 40 mm above the medial epicondyle and only in 10% (2/20) at its level by Sunderland [37] and in 10.81% (4/37) by Marur et al. In one case (2.70%) the branch arose 12 mm above the epicondyle level [24]. Gonzalez et al. [15] found this branch leaving the ulnar nerve above the epicondylar level in 5.13% (2/39), Paulos et al. [31] in 20% (4/20), and Ng et al. [29] in 30% (3/10). Two case reports describing such variable branch complicating surgery of the ulnar nerve exist to our knowledge [8, 19]. This first branch of the ulnar nerve supplies the FCU in 90%, the flexor digitorum profundus muscle in 5%, and both these muscles in 5% of cases [37]. Knowledge and subsequent protection of all motor branches are of the highest importance during mobilization of the ulnar nerve distal to the medial epicondyle of the humerus, as in the case of olecranon osteotomy or ulnar nerve transposition.

Another structure at risk is the posterior branch of the medial antebrachial cutaneous nerve, which is prone to injury especially during the cubital tunnel release as it crosses superficially the ulnar nerve. Lowe et al. [23] described that one to four branches may cross the ulnar nerve in 27.84%, 59.79%, 8.25%, and 4.12% percent, respectively, on the sample of 97 patients.

### Variants in deep dissection

**Fig. 2:**
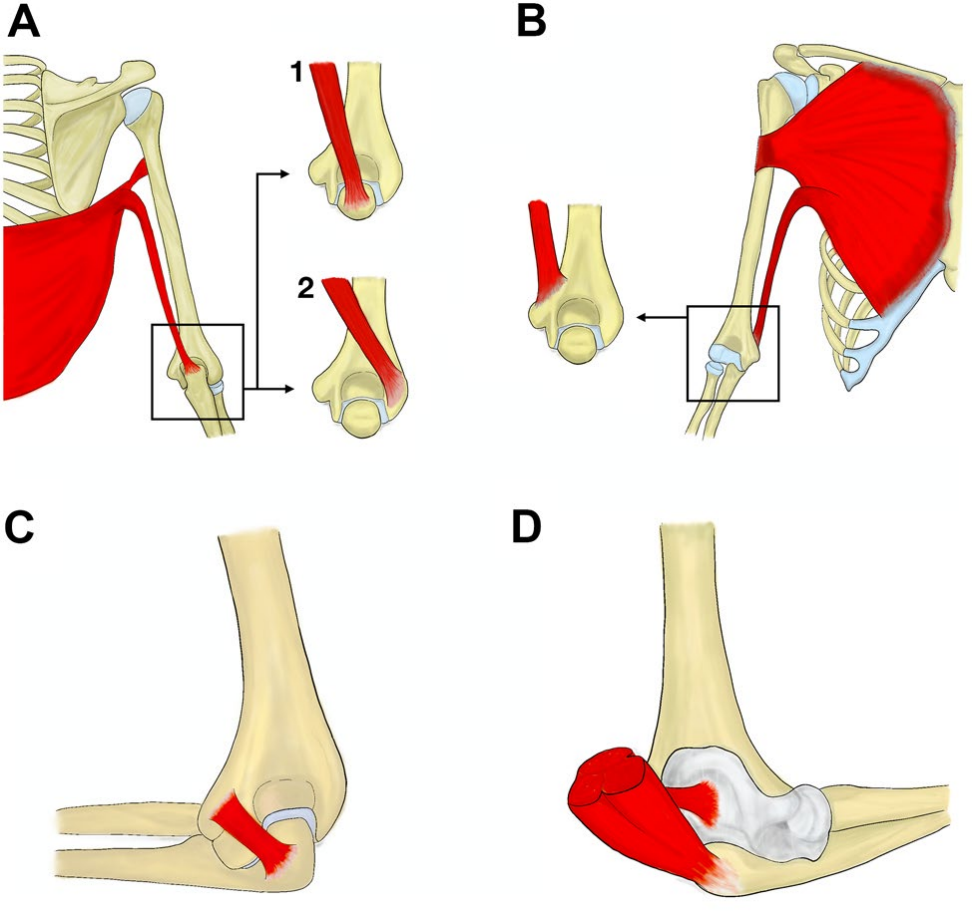
Origins and insertions of four described variable muscles around the elbow joint—dorsoepitrochlearis muscle **a** may insert on (1) lateral epicondyle or (2) olecranon, chondroeptirochlearis muscle, **b** usually inserts on medial epicondyle. Epitrochleoanconeus muscle, **c** originates from medial epicondyle and inserts on olecranon. Subanconeus muscle, **d** inserts on the dorsal aspect of the joint capsule

Then, the dissection is very straightforward, and the orientation is undemanding with the triceps brachii muscle serving as an impossible-to-miss landmark. The amount of dissection depends on the technique used to access the articular surface of the elbow joint. Several variable muscles were described in this area that cannot only confuse junior surgeons during the approach but can lead to clinical symptoms as well. Variable muscles attached to the olecranon are most significant during the posterior approach as they can limit its retraction when using the chevron osteotomy. Four such muscles were described in the available literature (Fig. 2).

Dorsoepitrochlearis muscle (latissimocondyloideus muscle) occurs in about 1.6–10% of individuals. It arises from the latissimus dorsi muscle and may insert on the olecranon, brachial fascia, triceps brachii muscle, shaft of the humerus, or the lateral epicondyle of the humerus [16, 40]. Although it is thought to be innervated by the thoracodorsal nerve, earlier reports mention the innervation by radial nerve as well [12, 16]. This muscle may rarely even limit the motion in the shoulder joint and is visible and palpable in the axilla [28].

Another aberrant muscle originates from the sternocostal part of the pectoralis major muscle, which is named the chondroepitrochlearis muscle. Its course is very similar to the dorsoepitrochlearis muscle, but it inserts on the medial epicondyle of the humerus, where it was described to cause ulnar nerve entrapment [36].

Anconeus epitrochlearis muscle (epitrochleoanconeus muscle) is present approximately in 1–29% of individuals but its occurrence differs significantly between authors [17, 27]. The muscle was found in 28.66% (45/157) [2], and 5.21% (5/96) in Japanese [27], and in 1% (1/100) [9] and 17.86% (10/56) [25] in German population. It is a short muscle, originating from the medial epicondyle of the humerus adjacent to the triceps brachii muscle and inserting on the olecranon. It is innervated by the ulnar nerve and may produce ulnar nerve compression [17, 40]. Wachsmuth reported 16 cases solved by myotomy and anterior transfer of ulnar nerve [43].

Subanconeus muscle (also known as articularis cubiti muscle or Lecomte’s pronator of ulna) was a gracile constant capsular muscle under the triceps brachii muscle. Nevertheless, nowadays many authors disagree that it is a separate muscle. It arises from the medial head of the triceps brachii muscle and inserts dorsally on the elbow joint capsule [39]. It was present to some extent in every specimen dissected by Tubbs et al. [41] (18/18) with varying number of muscular fascicles, but its function is still unclear.

**Fig. 3:**
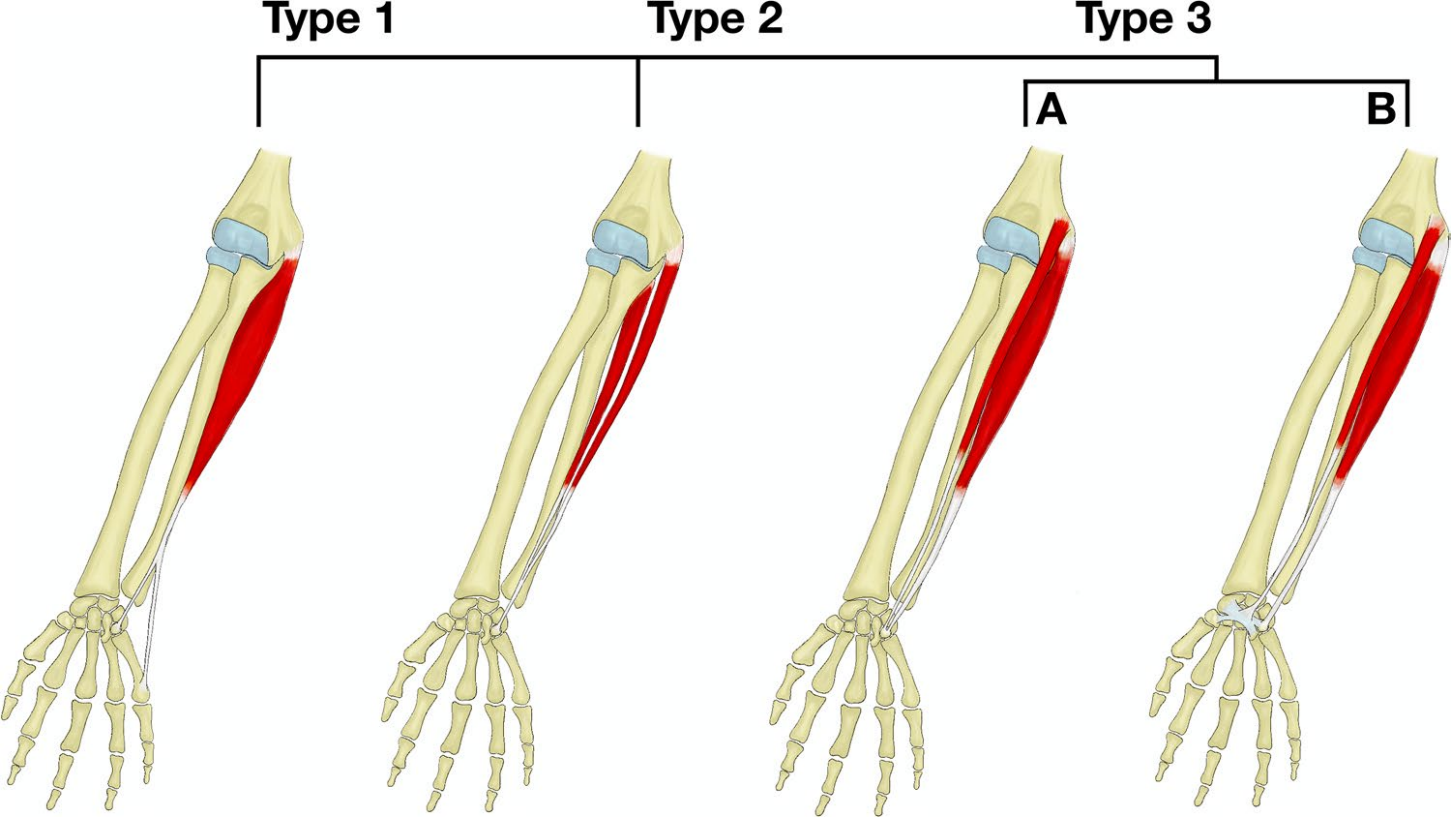
Modified Bhadarwaj’s classification of the FCU muscle variations **a** type I: single muscle with two tendons, **b** type II: two heads forming separate muscles and type III: completely accessory muscle with **c** “FCU-like” insertion and **d** “PL-like” insertion

During the triceps-splitting approach, the accessory FCU muscle may be spotted. Accessory ulnar muscles can be classified into four subunits and their presence should always raise caution as other variable structures are often concomitant. Type I is a single muscle with a split insertional tendon. In type II the heads of the FCU form two separate muscles, which fuse close to their insertion [3]. Nevertheless, most confusion may arise from types IIIA and IIIB, which are true separate accessory muscles attaching as “FCU-like” and “palmaris longus-like” insertions, respectively (Fig. 3) [21]. Although many relatively common variants of the flexor digitorum superficialis and extensor digitorum muscles have been described, they usually occur more distally in the forearm and therefore are not significant for the posterior approach [27, 40]. Osborne’s band is a variable structure occurring between the two head of the FCU. Its proper definition is still missing and therefore the exact prevalence is unknown [39]. It was defined as connective tissue between two heads of the FCU and reported to be present between 73% (49/64) [10] and 100% (39/39) [15]. Another possible definition is a connective tissue between the medial epicondyle and olecranon, which was reported to be present in 70–81% but other definitions exist as well [38]. More proximally the arcade of Struthers can be found, defined as a thickening of the brachial fascia expanding between the medial intermuscular septum of the arm and the medial head of the triceps brachii muscle. Its prevalence highly differs from 13.5% (8/60) [34] to 100% (40/40) [4] and it was also reported to cause ulnar nerve entrapment.

An accessory bone, the tricipital sesamoid bone (*os sesamoideum tricipitale*), can be rarely present within the triceps brachii muscle tendon in 0.05% (1/1940) [20]. This sesamoid bone may be quite large and limit the surgical division of the triceps brachii muscle tendon. Patella cubiti is another term used for this variant, which can often be symptomatic. Many different treatments were tried with differing results. Mittal et al. [26] recommend avoiding surgical intervention in patients presenting only with a stiff elbow.

### Intra-articular variations

**Fig. 4:**
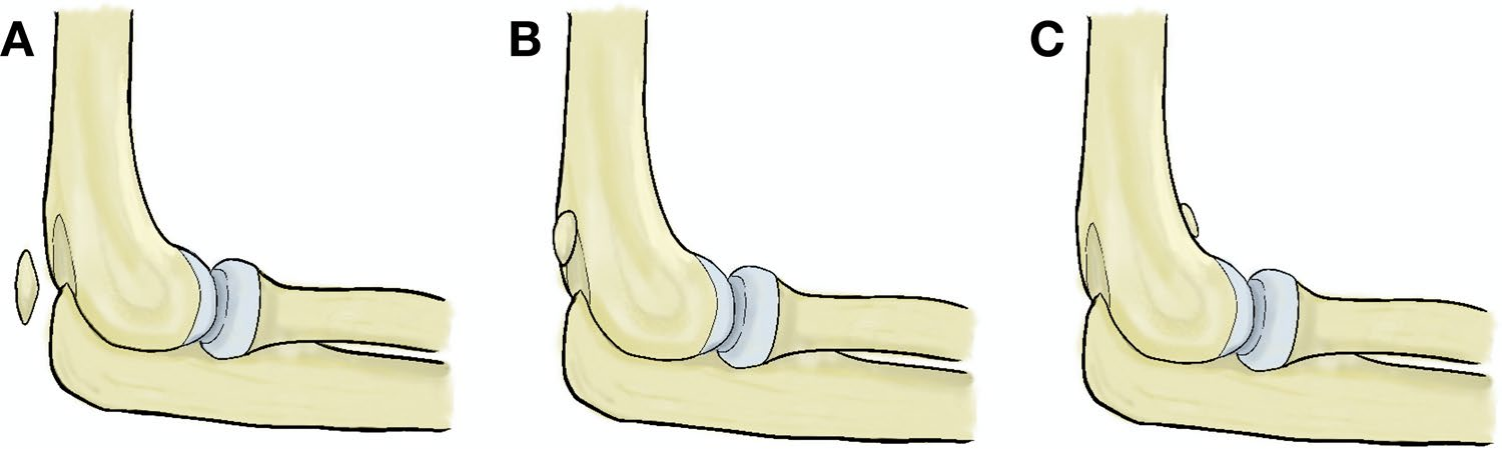
Three relevant described accessory bones encountered around the elbow joint—**a** tricipital sesamoid bone, **b** posterior supratrochlear bone, and **c** anterior supratrochlear bone

Other rare accessory bones, posterior and anterior supratrochlear bone (*os supratrochleare posterius et anterius*), may be encountered intra-articulary in the elbow joint [40, 41]. Such cases are often symptomatic and need surgical intervention [1]. Around 30 cases of the posterior supratrochlear bone are reported in the literature (Fig. 4) [45].

**Fig. 5:**
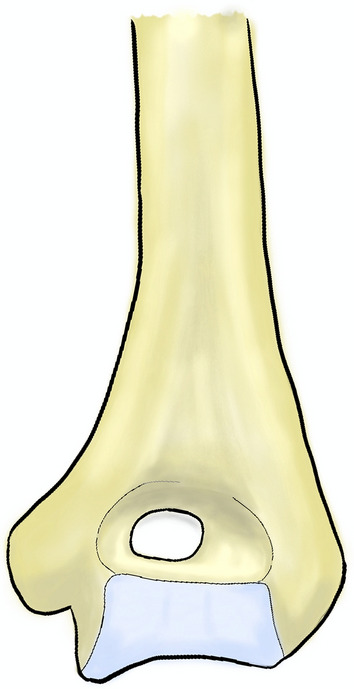
The supratrochlear foramen

During the visualization of the intra-articular surface a supratrochlear foramen can be noticed (Fig. 5). Its prevalence ranges from 0.3% in Greeks to 58% in Arkhan Indians, respectively [13]. Clinical significance consists of a predisposition to the distal humerus fracture and decreased availability of space for surgical hardware [32, 33].

## Conclusion

Many variants can be encountered in the area of the elbow joint and their knowledge is essential to truly understand its anatomy. The presented review enables easier orientation in the current literature with the aim on the posterior approach towards the elbow joint. A complex study describing the prevalence of the anatomical variants, which can be met during this approach, is still missing and would provide further information about their importance.

## Data Availability

All data that support findings of the current study are accessible upon reasonable request from the corresponding author.
